# The Effectiveness of Remote Consultations During the COVID-19 Pandemic: A Tool for Modernising the National Health Service (NHS)

**DOI:** 10.7759/cureus.32301

**Published:** 2022-12-07

**Authors:** Clara Smith, Bara Kubanova, Fahid Ahmed, Jai Manickavasagam

**Affiliations:** 1 Plastic and Reconstructive Surgery, Ninewells Hospital, Dundee, GBR; 2 Otolaryngology - Head and Neck Surgery, Ninewells Hospital, Dundee, GBR

**Keywords:** healthcare technology, otolaryngology, telephone consultations, video consultations, remote consultations

## Abstract

Objective

The coronavirus disease 2019 (COVID-19) pandemic prompted major changes to the delivery of care. There was a move towards remote consultations in order to mitigate the risk of viral exposure and the risk of delaying care. Remote consultations will play a prominent role within the National Health Service (NHS) in the future. This project aimed to evaluate the effectiveness of remote consultations relative to face-to-face (F2F) consultations.

Methods

A local retrospective audit of remote consultations in ENT was performed by comparing outcome data for video and telephone appointments during the first peak of the pandemic to outcomes for F2F consultations during the same months of the preceding year. Chi-square tests were employed to determine whether there was any statistically significant discrepancy between the two modalities.

Results

Outcomes from a total of 314 patient consultations were reviewed. One hundred and fifty-four patients were male, and 160 were female; 111 patient consultations were conducted F2F, and 203 remotely (101 via telephone and 102 via video). There was no statistically significant difference detected between remote and F2F groups for rates of investigation, listing for theatre, referral to other specialties, and initiating treatment. Patients reviewed remotely were less likely to be discharged than those reviewed F2F (p=<0.001). Comparing the two remote modalities, telephone patients were more likely to undergo investigation than patients reviewed over video (p = 0.031).

Conclusions

Remote consultations were an effective and reliable resource for maintaining a high standard of care during the COVID-19 pandemic. Our findings suggest that remote consultations will prove a valuable tool for clinicians in the remobilisation of health services in the post-pandemic era.

## Introduction

On March 11, 2020, the WHO declared the coronavirus disease 2019 (COVID-19) outbreak as a global pandemic. Healthcare systems around the world were overwhelmed by the volume of critically ill patients requiring life-supporting treatment. The situation in the United Kingdom was no exception; the pandemic had drastic implications for the day-to-day delivery of services and exacerbated existing weaknesses within the National Health Service (NHS), including waiting list times. 

In 2022, the pandemic continues to present a burden and, as the current waiting list crisis shows no sign of abating, it is vital for clinicians to do their utmost to support the integration of new technologies aimed at tackling existing constraints. Clinical analysis of the effectiveness of these technologies is an essential step in the process of ensuring standards are upheld and patient safety is maintained.

The unprecedented disruption caused by the arrival of the COVID-19 pandemic served as a catalyst for the adoption of novel technologies that had, hitherto, experienced poor uptake. Within NHS Tayside, there was a rapid shift to predominantly virtual consultations as a necessary means for continuing service provision and protecting vulnerable patients and staff. This presented an invaluable opportunity to analyse the effectiveness of virtual consultations in ear, nose, and throat (ENT) surgery within our local department. This was achieved by comparing virtual outcomes to the outcomes of face-to-face (F2F) consultations. We are not aware of a prior study that directly compares the two. 

## Materials and methods

After obtaining the Caldicott approval, a local retrospective audit of virtual consultations was performed by analysing outcome data from telephone and video consultations conducted during the peak of the first wave of the COVID-19 pandemic. Video consultations were conducted using ‘Near Me Video Appointment' software. Patient allocation to either video or telephone clinics had been performed essentially at random, based on clinic availability.

As F2F consultations form the traditional method for assessment and delivery of care, outcome data collected from F2F consultations within the same period of the preceding year (i.e pre-COVID-19) was used to establish a standard of care. In addition to comparing virtual consultations to F2F, each remote modality was then studied in turn to determine whether there was any significant difference between the outcomes for video and telephone consultations.

Statistical analysis was performed using IBM SPSS Statistics for Windows, Version 27.0 (Released 2020; IBM Corp., Armonk, New York, United States). Univariate analysis was run, creating a general linear model, with independent variables being the consultation modality, and the dependent variables being the effectiveness measures. Effectiveness measures were defined by the following consultation outcomes: added to theatre list, investigation requested, treatment initiated, onward referral made, and discharged.

Further data were collected for a number of covariates that had the potential to influence the consultation outcome. These were: clinician, subspecialty, diagnosis, whether the consultation was for a new or follow-up patient, and whether the patient was from the paediatric or adult population. Where outcomes of virtual consultations were comparable to those for F2F consultations, i.e. where there was no significant variation, it could be concluded that the modality was effective in maintaining the standard of care. 

Our findings have been presented in line with the Standards for Quality Improvement Reporting Excellence (SQUIRE) 2.0 reporting method.

## Results

**Figure 1:**
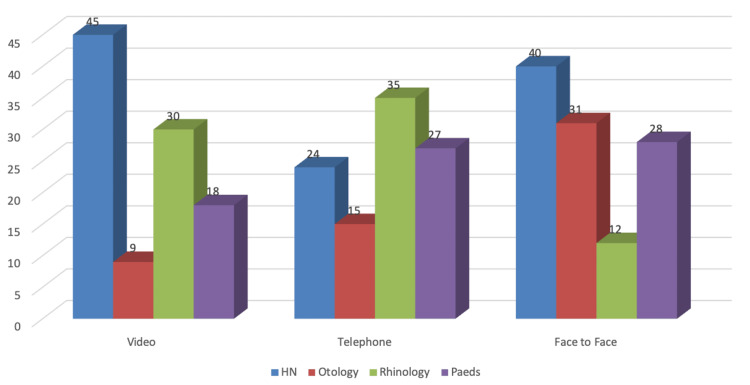
Number of presentations by subspecialty for each modality Paeds: paediatrics; HN: head and neck

Outcomes from a total of 314 patient consultations were reviewed. One hundred and fifty-four patients were male and 160 were female. The age distribution of the cohort was 6-89 years, giving a mean age of 42 years, and a median age of 47 years within the patient population, revealing a negatively skewed distribution. One hundred and eleven patient consultations were conducted F2F, and 203 remotely. Of the virtual consultations, 101 were via telephone and 102 via video (Figure 1). In only 3.9% of video and 5.0% of telephone consultations was it not possible to safely assess or manage the presenting complaint virtually and, therefore, the patient was reviewed in person at the next available F2F clinic.

Virtual vs F2F consultations

Comparing outcomes between virtual and F2F consultations revealed largely similar results (Table 1). Of patients reviewed F2F, 20.7% subsequently underwent investigation, versus 22.2% in the virtual category (p = 0.766); 12.6% of F2F consultations resulted in patients being listed for theatre compared to 6.4% from the virtual category (p = 0.061). Of the 111 patients seen in person, 3.6% were referred to another specialty. This did not vary significantly from the 4.4% of patients referred from the virtual group (p = 0.724). Patients were as likely to commence treatment whether they were reviewed F2F or virtually (25.2% and 24.6%, respectively, p = 0.907). The first significant finding was that patients reviewed remotely were less likely to be discharged than patients reviewed F2F (17.2% vs 45.9%, respectively; p = <0.001).

**Table 1:** Outcomes of virtual vs face to face (F2F) consultations

Outcome	Virtual (%)	F2F (%)	p-value
Added to theatre list	6.4	12.6	0.061
Investigation requested	22.2	20.7	0.766
Treatment initiated	25.2	24.6	0.907
Onward referral made	4.4	3.6	0.724
Discharged	17.2	45.9	0.001

Virtual modalities compared: video vs telephone

Comparing outcomes for the two virtual modalities (Table 2) revealed a statistically significant discrepancy in the rates of performing investigations, with telephone patients more likely to undergo investigation than video consults (84.2% vs 71.6%, p = 0.031). All other outcomes between the telephone and video groups were similar, including listing for theatre (5.9% vs 6.9%, p = 0.788), onward referrals (5.9% vs 2.9%, p = 0.299); initiating treatment (21.8% vs 27.5%, p = 0.349), and discharge rates (20.8% vs13.7%, p = 0.183).

**Table 2:** Virtual modalities compared: video vs telephone

Outcome	Video (%)	Telephone (%)	p-value
Added to theatre list	6.9	5.9	0.788
Investigation requested	71.6	84.2	0.031
Treatment initiated	27.5	21.8	0.349
Onward referral made	2.9	5.9	0.299
Discharged	13.7	20.8	0.183

Impact of covariates

There was a significant discrepancy between clinicians in initiating treatment in virtual consultations (p = 0.001). The reviewing clinician was also a significant factor in determining how likely a patient was to be investigated or listed for theatre in the F2F category (p = 0.047 and p = 0.012, respectively). 

There was a statistically significant difference in initiating treatment across different subspecialties; this was the case in both virtual (p = <0.001 ) and F2F (p = 0.008) consultations. There was a significant difference in investigation rates depending on the diagnosis in the virtual group only (p= 0.002). In both virtual and F2F groups, the diagnosis had a significant impact on treatment initiation (p = <0.001 and 0.034, respectively).

New patients were more likely than follow-up patients to be investigated in both the virtual setting (p = 0.009) and F2F (p = 0.02). Initiation of treatment was more likely in new patients in both settings (virtual, p = 0.027; in person, p = 0.005). New patients were more likely than follow-up patients to be referred to other specialties in the virtual group (p = 0.023).

Adult and paediatric patients were equally as likely to receive treatment F2F (p=0.096). When reviewed virtually, however, adult patients were significantly more likely to receive treatment compared to paediatric patients (p=0.017). Paediatric patients were statistically more likely to be listed for theatre than adults in the in-person group (p = 0.003). This pattern was not observed in the remote group, where there was no significant difference. 

## Discussion

The move towards the incorporation of virtual technology into healthcare provision pre-dates the pandemic [1]; safely harnessing its potential will improve access to specialty care and support the sustainability of the NHS in the 21st century [2]. The unique conditions presented by the pandemic, and the accompanying disruption it created, served as the necessary catalyst for the adoption of change [3].

To patients, virtual consultants offer some favourable advantages over F2F review: the technology offers a means of overcoming geographical barriers and eliminating the financial cost associated with transport and parking [4]. This is particularly true for patients living in rural areas where access to secondary care is challenging for practical reasons. In a similar vein, it also presents a welcome alternative for the elderly and patients with disabilities. Of course, the ability to participate in virtual consultation is dependent upon the ability to access and interact with, the appropriate technology, an obvious limiting factor. Virtual consultations have also been proposed as a more cost-effective means of providing care for health service, particularly in the setting of the long-term management of chronic illness [5].

Previous studies have demonstrated that in the right setting, virtual consultations are both a safe method for the delivery of care and popular with clinicians and patients [6]. However, further research is required to outline exactly what constitutes 'the right setting', including which specialties or conditions are most suitable for virtual review. The advantages and limitations of the use of virtual technology in a number of other specialties have previously been examined [5], but data specific to ENT is sparse. Lesser still, is literature that includes an assessment of the use of video technology. 

A small study conducted by an ENT department in Wales demonstrated that virtual consultations, including video, could be successfully implemented in ENT, and served as a useful adjunct to standard F2F appointments [7]. However, it did not attempt to compare outcomes between virtual and F2F modalities.

As far as the authors are aware, this is the first paper to compare the outcomes of virtual consultations to the standard practice of F2F consultations. Our study demonstrated no significant variation in rates for listing for theatre, ordering investigations, initiating treatment, or referring to other specialties between the virtual and F2F groups. Those patients listed for theatre would still be required to undergo consent for surgery F2F at some stage prior to surgery, in accordance with the principles of ethics outlined in the General Medical Council (GMC) good medical practice recommendations [8].

Clinicians were significantly less likely to discharge patients from virtual consultations than they were after reviewing patients F2F. The limited potential for examination may be a factor undermining physician confidence when it comes to discharging patients from the virtual setting. Indeed, an evaluation of clinicians’ experience of using virtual consultations during the COVID-19 pandemic reported that the primary complaint regarding virtual consultations was the inability to physically examine patients; this was considered particularly important by those working in surgical specialties [9]. In the F2F setting, the physical examination is typically considered an essential step in formulating a differential diagnosis and management plan.

Patients reviewed by telephone were significantly more likely to be investigated than those reviewed via video; perhaps, related to an increased capacity for examination when using video rather than audio alone. This would be consistent with the literature, which reports greater diagnostic and decision-making accuracy when using video technology rather than the telephone [10].

Other significant findings in our report were rather predictable; one would expect, for example, that the initiation of treatment would vary with subspecialty and diagnosis. Similarly, it is intuitive that new patients are more likely to be investigated or commenced on treatment when compared to follow-up patients. 

It is interesting, however, that clinicians appeared more reluctant to initiate treatment in the paediatric population without F2F review. In addition, the trend of paediatric patients being more likely than adult patients to require theatre disappeared in the remote population. This, perhaps, suggests that paediatric otolaryngology is a subspecialty less well suited to virtual consultation. 

The disparity in behaviour amongst clinicians using virtual technology could in part be attributable to variable familiarity levels; however, it is likely to be partly explained by variation in clinician subspecialty. This study did not investigate clinicians’ prior experience of using the technology and, therefore, it cannot be determined whether this was a factor or not. Clinician subspecialty is certainly the most likely explanation for differences in investigation rates and theatre rates amongst clinicians in the F2F setting.

In an independent United Kingdom study, clinicians reported concerns regarding the medicolegal implications of reviewing patients virtually, including via video [9]. In a United States review of malpractice suits lodged following a dissatisfactory telephone consultation, the most common allegation was failed diagnosis [11]. There is, however, evidence to suggest virtual consultations provide a reliable means of assessing ENT patients: in a United States study of 250 patients with laryngology-related complaints, overall concordance rates for diagnosis and management between the initial telephone-based review and subsequent physical examination with laryngoscopy were high at 86.1% and 93.7%, respectively [12]. Conducting research aligned with the United States study here in the United Kingdom may help to enhance clinicians’ confidence in their virtual decision-making. Further studies have gathered qualitative and/or quantitative information on the efficacy of telephone consultations within individual ENT departments, with high levels of patient and clinician satisfaction reported [13-15].

Limitations

A recognised limitation of our study is the small sample size. Further research on a larger scale is recommended to substantiate the findings contained within this report and elucidate which conditions are more, or less, well suited to being managed remotely. Furthermore, we did not investigate rates of re-presentation following discharge from virtual clinics. This study did not attempt to calculate the effective cost saving of virtual technology over F2F review. ‘Did not attend’ data was not collected and nor was a patient satisfaction survey conducted.

We also recognise the potential for subspecialty and consultant-level bias in the form of technology familiarity and a variable learning curve. The collection of qualitative data used to examine the experience of clinicians, their concerns, and any ideas around perceived limitations of the technology would also be of benefit.

## Conclusions

Prior to this research, we hypothesised that virtual consultations would prove an effective alternative for supporting the delivery of healthcare. We endeavoured to analyse performance against that of the standard practice of F2F consultations and demonstrate whether or not the outcomes were comparable. We anticipated that some variability between users and subspecialties may exist. This study demonstrates that outcomes for telephone and video modalities are largely similar to those for F2F review. It also highlights some discrepancies in decision-making between different modalities, as well as subspecialties that are perhaps less well suited to remote review, such as paediatric otolaryngology. Reproduction of this study with a larger sample size would help to validate these findings and reveal more information on the suitability of specific conditions. Further qualitative research that aims to understand the beliefs and/or factors underpinning the differences in clinician behaviour between modalities would also be of benefit. The use of virtual technology within the NHS remains in its infancy, presenting numerous avenues for further research; however, the results of this study support the conclusion that virtual technology is a valuable asset to the NHS. 
